# Inhibition of ChREBP ubiquitination via the ROS/Akt-dependent downregulation of Smurf2 contributes to lysophosphatidic acid-induced fibrosis in renal mesangial cells

**DOI:** 10.1186/s12929-022-00814-1

**Published:** 2022-05-10

**Authors:** Donghee Kim, Ga-Young Nam, Eunhui Seo, Hee-Sook Jun

**Affiliations:** 1 Lee Gil Ya Cancer and Diabetes Institute, Gachon University, 155, Gaetbeol-ro, Yeonsu-gu, Incheon, 21999 Republic of Korea; 2 College of Pharmacy and Gachon Institute of Pharmaceutical Sciences, Gachon University, 191, Hambangmoe-ro, Yeonsu-gu, Incheon, 21936 Republic of Korea; 3 Gachon Medical Research Institute, Gil Hospital, 21, Namdong-daero 774beon-gil, Namdong-gu, Incheon, 21565 Republic of Korea

**Keywords:** Diabetic nephropathy, Mesangial cells, Fibrosis, Lysophosphatidic acid, ChREBP, Smurf2

## Abstract

**Background:**

Mesangial cell fibrosis, a typical symptom of diabetic nephropathy (DN), is a major contributor to glomerulosclerosis. We previously reported that the pharmacological blockade of lysophosphatidic acid (LPA) signaling improves DN. Although LPA signaling is implicated in diabetic renal fibrosis, the underlying molecular mechanisms remain unclear. Here, the role of carbohydrate-responsive element-binding protein (ChREBP) in LPA-induced renal fibrosis and the underlying mechanisms were investigated.

**Methods:**

Eight-week-old wild-type and db/db mice were intraperitoneally injected with the vehicle or an LPAR1/3 antagonist, ki16425 (10 mg/kg), for 8 weeks on a daily basis, following which the mice were sacrificed and renal protein expression was analyzed. SV40 MES13 cells were treated with LPA in the presence or absence of ki16425, and the expression of ChREBP and fibrotic factors, including fibronectin, TGF-β, and IL-1β, was examined. The role of ChREBP in the LPA-induced fibrotic response was investigated by ChREBP overexpression or knockdown. The involvement of Smad ubiquitination regulatory factor-2 (Smurf2), an E3 ligase, in LPA-induced expression of ChREBP and fibrotic factors was investigated by Smurf2 overexpression or knockdown. To identify signaling molecules regulating Smurf2 expression by LPA, pharmacological inhibitors such as A6370 (Akt1/2 kinase inhibitor) and Ly 294002 (PI3K inhibitor) were used.

**Results:**

The renal expression of ChREBP increased in diabetic db/db mice, and was reduced following treatment with the ki16425. Treatment with LPA induced the expression of ChREBP and fibrotic factors, including fibronectin, TGF-β, and IL-1β, in SV40 MES13 cells, which were positively correlated. The LPA-induced expression of fibrotic factors increased or decreased following ChREBP overexpression and knockdown, respectively. The production of reactive oxygen species (ROS) mediated the LPA-induced expression of ChREBP and fibrotic factors, and LPA decreased Smurf2 expression via Traf4-mediated ubiquitination. The LPA-induced expression of ubiquitinated-ChREBP increased or decreased following Smurf2 overexpression and knockdown, respectively. Additionally, Smurf2 knockdown significantly increased the expression of ChREBP and fibrotic factors. The pharmacological inhibition of Akt signaling suppressed the LPA-induced alterations in the expression of ChREBP and Smurf2.

**Conclusion:**

Collectively, the results demonstrated that the ROS/Akt-dependent downregulation of Smurf2 and the subsequent increase in ChREBP expression might be one of the mechanisms by which LPA induces mesangial cell fibrosis in DN.

**Supplementary Information:**

The online version contains supplementary material available at 10.1186/s12929-022-00814-1.

## Background

Diabetic nephropathy (DN) is a major diabetic microvascular complication, and is a common cause of end-stage renal disease, worldwide [1]. The major pathological features of DN include glomerular hypertrophy, increased production and accumulation of extracellular matrix, mesangial expansion, thickening of the basement membrane, loss of glomerular filtration function, and renal fibrosis, which results in the progressive loss of renal function [2, 3]. In particular, the mesangial expansion leading to renal fibrosis is due to the proliferation of mesangial cells, which are one of the major types of resident renal cells, and the excessive accumulation of extracellular matrix [4]. Therefore, a better understanding of the mechanisms that induce fibrosis in renal mesangial cells would aid the development of drugs for suppressing the progression of DN in the clinic.

Lysophosphatidic acid (LPA) is a phospholipid derivative that acts as a signaling molecule in various cellular functions, including proliferation, survival, and migration, via six G protein-coupled receptors (GPCRs; LPA receptor (LPAR) 1–6) [5, 6]. LPA can induce the proliferation and pro-fibrotic response in numerous types of cells, including renal mesangial cells [7–9]. We have previously reported that LPA induces fibrotic response in renal mesangial cells [10]. We also observed that the inhibition of LPA/LPAR1 signaling inhibits renal fibrosis and improves DN in type 2 db/db diabetic mice [10] and mice with streptozotocin (STZ)-induced diabetic mice [11]. Despite implication of LPA in diabetic renal fibrosis, the underlying molecular mechanisms are not fully defined.

The carbohydrate-responsive element-binding protein (ChREBP) protein is a basic helix–loop–helix leucine zipper (bHLH-LZ) transcription factor that plays an important role in glucose and lipid metabolism [12, 13]. ChREBP is mainly expressed in the liver and white and brown adipose tissues, and is also moderately expressed in the intestine, skeletal muscles, and kidneys [14]. High levels of glucose and advanced glycation end products (AGEs), which are diabetic factors, promote the expression of ChREBP and cell proliferation by increasing the production of reactive oxygen species (ROS) in hepatic cancer cells [15]. It has been reported that high glucose-induced O-GlcNAcylated ChREBP mediates lipogenesis and fibrosis in mesangial cells [16]; however, the role of ChREBP in the LPA-induced fibrotic response involved the development of DN remains incompletely understood.

The aim of this study was to investigate the role of ChREBP in LPA-induced renal fibrosis, and elucidate the underlying mechanisms. We observed that the ROS/Akt-dependent downregulation of Smad ubiquitination regulatory factor-2 (Smurf2) and the subsequent increase in the expression of ChREBP via the inhibition of ubiquitination could be one of the mechanisms underlying the LPA-induced fibrosis of renal mesangial cells in DN.

## Materials and methods

### Experimental animals

Eight-week-old male diabetic db/db (BKS.Cg-lepr^db^/lepr^db^) and non-diabetic wild-type (BKS.Cg-lepr^+^/lepr^+^) mice on the C57BLKS/J background were purchased from Jackson Laboratories (Bar Harbor, ME, USA). The wild-type mice were used as the age-matched controls. Ki16425 (Biorbyt Ltd., Cambridge, UK) or the vehicle was intraperitoneally injected at a dose of 10 mg/kg for 8 weeks on a daily basis. After removing the renal capsule, the right kidney was fixed in formalin, and the renal cortex was dissected from the left kidney under a dissecting microscope. All the animal experiments were approved by the Institutional Animal Care and Use Committee at the Lee Gil Ya Cancer and Diabetes Institute, Gachon University (LCDI-2016-0080).

### Cell culture and treatment

SV40-transformed murine glomerular mesangial cells (SV40 MES13) were obtained from the American Type Culture Collection (CRL-1927, Rockville, MD, USA) and maintained in a 3:1 mixture of DMEM (Welgene, Gyeongsangbuk-do, Korea) and F-12 medium (Life Technologies, Grand Island, NY, USA) supplemented with 5% fetal bovine serum (FBS, Life Technologies), 14 mM HEPES (Life Technologies), and 1% penicillin–streptomycin (Welgene Inc.) at 37 °C in an atmosphere of 5% CO_2_ and 95% air.

In order to investigate the effect of LPA, the SV40 MES13 cells (3 × 10^5^ cells) were plated on 60 mm dishes and cultured for 24 h. Based on our previous report [10], the medium was replaced with serum-free medium (SFM) containing 0.1% fatty acid-free bovine serum albumin (FAF-BSA, Sigma-Aldrich, St. Louis, MO, USA), and incubated for 16–18 h. The cells were then treated with 10 μM LPA (18:1 Lyso PA (1-oleoyl-2-hydroxy-sn-glycero-3-phosphate); Avanti Polar Lipids, Birmingham, AL, USA) in the presence or absence of 10 μM ki16425 (Biorbyt Ltd.).

In order to determine the involvement of ROS, the cells were pretreated with 10 mM N-acetyl-l-cysteine (NAC; Sigma-Aldrich) for 1 h, followed by treatment with 10 μM LPA for 3 h or 100 μM H_2_O_2_ for 1 h. For inhibition of Akt signaling, the cells were treated with 4 µM A6730 (Akt1/2 kinase inhibitor; Sigma-Aldrich) or 10 µM Ly 294002 (2–4-morpholinyl-8-phenlchromone, PI3K inhibitor; Sigma-Aldrich) for 1 h prior to LPA treatment. The activity of Smurf2 was inhibited by treating the cells with 50 µM Heclin (an inhibitor of a homologous to the E6-AP carboxyl terminus (HECT)-type E3 ubiquitin ligases; Sigma-Aldrich) for 3 h.

### Immunohistochemical and immunocytochemical staining

Following fixation with 10% neutral buffered formalin (Sigma-Aldrich), the kidney tissues were embedded in paraffin and sliced into 4 µm-thick sections. The sections of kidney tissue were subjected to heat-induced antigen retrieval in citrate buffer (10 mM citric acid and 0.05% Tween 20; pH 6.0) and permeabilized using 0.2% Triton X-100 in PBS for 10 min. The sections were then blocked in Protein Block Serum-Free Ready-To-Use solution (Dako North America, Inc., Carpinteria, CA, USA) at 25 °C for 1 h. The sections were subsequently incubated overnight at 4 °C with ChREBP (NB400-135; Novus Biologicals, Littleton, CO, USA) or Smurf2 (#12024; Cell Signaling Technology (CST), Boston, MA, USA) antibodies diluted at 1:200 in antibody diluent (Dako North America, Inc.). For the detection of ChREBP, the sections were incubated with anti-rabbit Alexa 546 (A11010; Invitrogen; Thermo Fisher Scientific, Inc., MA, USA) secondary antibody and anti-actin, α-smooth muscle (α-SMA)-FITC antibody (1A4 clone; F3777; Sigma-Aldrich) at a dilution of 1:200. The nuclei were stained with 4,6-diamidino-2-phenylindole (DAPI; Invitrogen, Carlsbad, CA, USA) at a dilution of 1:1000 in PBS, and mounted with a fluorescence mounting medium (Dako North America, Inc.). The sections were observed using a confocal microscope (Carl Zeiss Inc., Oberkochen, Germany). For the detection of Smurf2, the tissues were stained with Polink-2 Plus HRP Rabbit with DAB Kit (D39-18, GBI Labs. Inc., Bothell, DC, USA) according to the manufacturer’s protocol. The nuclei were counterstained with hematoxylin. Immunohistochemical analysis was performed using a confocal microscope LSM 700 (Carl Zeiss Inc.) and light microscope AXIO Imager Z1 (Carl Zeiss Inc.) at Core-facility for Cell to In-vivo imaging.

For immunocytochemical staining of ChREBP, SV40 MES13 cells (2 × 10^4^ cells/well) were seeded in a 4-well chamber, and cultured for 18 h. The medium was replaced with SFM containing 0.1% FAF-BSA and the cells were incubated for 16–18 h, following which the cells were treated with 10 μM LPA in the presence or absence of 10 μM ki16425 for 3 h. The cells were fixed, permeabilized, blocked, and incubated overnight at 4 °C with a ChREBP antibody (NB400-135; Novus Biologicals) at a dilution of 1:200. The cells were subsequently incubated with an anti-rabbit Alexa Fluor 546 secondary antibody (A11010; Invitrogen) at a dilution of 1:200. The nuclei were stained with DAPI, mounted, and observed under a confocal microscope (Carl Zeiss Inc.).

### Quantitative real-time RT-PCR (qRT-PCR) analysis

The total RNA was extracted from the cells using RNAiso reagent (Takara Bio Inc., Kyoto, Japan), according to the manufacturer’s instructions. The cDNA was synthesized using a PrimeScript First Strand cDNA Synthesis Kit (Takara Bio Inc.). And qRT-PCR was performed using reaction mixture of SYBR Premix Ex Taq II (Takara Bio Inc.) in Real-Time PCR Sequence Detection System (StepOnePlus, Applied Biosystems, CA, USA), applying the following thermal protocol: a step at 50 °C for 2 min, an initial denaturation step at 95 °C for 2 min followed by 40 cycles of amplification (95 °C for 10 s, and 60 °C for 1 min). The relative gene expression levels were normalized to the expression levels of the 18s rRNA based on the Ct value. The sequences of the primer pairs used for PCR are enlisted in Additional file 1: Table S1.

### Western blotting

The tissues of the renal cortex were harvested, and the total protein extracts were obtained using M-PER™ Mammalian Protein Extraction Reagent (Thermo Fisher Scientific, Waltham, MA, USA), containing a protease inhibitor cocktail and a phosphatase inhibitor cocktail (Sigma-Aldrich). The extraction of the total cellular protein and western blotting were performed as previously described in our report [17], using primary antibodies against ChREBP (sc-21189; Santa Cruz Biotechnology, Santa Cruz, CA, USA; NB400-135; Novus Biologicals), Smurf2 (#12024; Cell Signaling Technology (CST), Boston, MA, USA), fibronectin (sc-8422; Santa Cruz Biotechnology; ab6328; Abcam, Cambridge, UK), TGF-β (#3711; CST), IL-1β (sc-52012; Santa Cruz Biotechnology), p-Akt1/2/3 (C-11) (sc-514032; Santa Cruz Biotechnology), Akt1/2/3 (5C10) (sc-81434; Santa Cruz Biotechnology), Traf4 (sc-136107; Santa Cruz Biotechnology), and β-actin (sc-47778; Santa Cruz Biotechnology; #8457; CST). The signals were detected using the ChemiDoc XRS + system, using the Image Lab software (Bio-Rad, Hercules, CA, USA) or a LAS-4000 mini system (Fujiflm Corp., Tokyo, Japan). The protein bands were quantified using ImageJ software (National Institutes of Health, Bethesda, MD, USA).

### Measurement of intracellular levels of ROS

SV40 MES13 cells (2 × 10^5^ cells/well) were seeded in 6-well plates and incubated for 24 h. The medium was replaced with SFM containing 0.1% FAF-BSA and incubated for 16–18 h. The cells were either treated with 10 μM LPA for different durations (0.5–2 h), or treated with 10 μM LPA in the presence or absence of 10 μM ki16425 for 1 h. The intracellular levels of ROS were measured by flow cytometry using 2,7-dichlorodihydrofluorescein diacetate (DCFH-DA, Invitrogen), as described in our previous report [18]. Flow cytometric analysis was done by using a FACSCalibur and LSR II (BD Biosciences) at Core-facility for Cell to In-vivo imaging.

### Transfection experiments

Murine ChREBP and Mlx expression vectors were kindly gifted by Dr. Howard C. Towle [19]. In order to induce the overexpression of ChREBP or Smurf2, SV40 MES13 cells were transiently co-transfected with ChREBP or Mlx expression vectors, or transfected with pCMV5B-Flag-Smurf2 wt (Addgene, Cambridge, MA, USA) or pCMV5B-Flag-Smurf2 C716A (catalytically inactive form of Smurf2; Addgene) using Lipofectamine 3000 (Invitrogen), according to the manufacturer’s instructions. For the small interfering RNA (siRNA) transfections, SV40 MES13 cells were plated and transiently transfected with ChREBP siRNA (Santa Cruz Biotechnology), non-specific double-stranded control siRNA (Santa Cruz Biotechnology), Smurf2 siRNA (Bioneer Inc., Daejeon, Korea), Traf4 siRNA (Bioneer Inc.), or scrambled siRNA (Bioneer Inc.) using Lipofectamine RNAiMAX (Invitrogen), according to the manufacturer’s instructions. After 6 h, the medium was replaced with SFM containing 0.1% FAF-BSA and incubated for 16–18 h, following which the cells were stimulated with 10 μM LPA for 3 h or left unstimulated.

### Immunoprecipitation studies

SV40 MES13 cells (2 × 10^5^ cells) were treated with 10 μM LPA in the presence or absence of 10 μM ki16425 for 3 h. In order to investigate the effect of Smurf2 expression on the ubiquitination of ChREBP, the cells were transiently transfected with Smurf2 siRNA or a Smurf2 overexpression vector, and subsequently treated with 10 μM LPA for 3 h. Following LPA treatment, the cells were rinsed twice with ice-cold PBS and subsequently lysed in lysis buffer (50 mM Tris pH 8.0, 150 mM NaCl, and 0.5% NP-40) supplemented with protease inhibitor cocktails (GenDEPOT Inc., Katy, TX, USA). The clear cell lysates (0.2–0.5 mg protein) were incubated for 3 h with 0.5 µg of ChREBP antibody (NB400-135; Novus Biologicals) or Smurf2 antibody (#12024; CST) with rotation. The mixtures were subsequently precipitated by mixing with 20 µL of protein A/G plus agarose (sc-2003; Santa Cruz Biotechnology) at 4 °C for 16–18 h. The beads were washed twice with 1 ml of DPBS and boiled in 2 × sample loading buffer for 10 min. The cell lysates and immunoprecipitates were analyzed by western blotting.

### Statistical analyses

The statistical analyses were performed by one-way analysis of variance (ANOVA) with Tukey’s multiple comparison test using GraphPad Prism version 7.03 (GraphPad Software Inc., San Diego, CA, USA). The data are presented as the mean ± standard error of the mean (SEM). Statistical significance was considered at p < 0.05.

## Results

### ChREBP protein expression decreased in the renal cortex of db/db mice treated with the LPAR1/3 inhibitor, ki16425

**Fig. 1:**
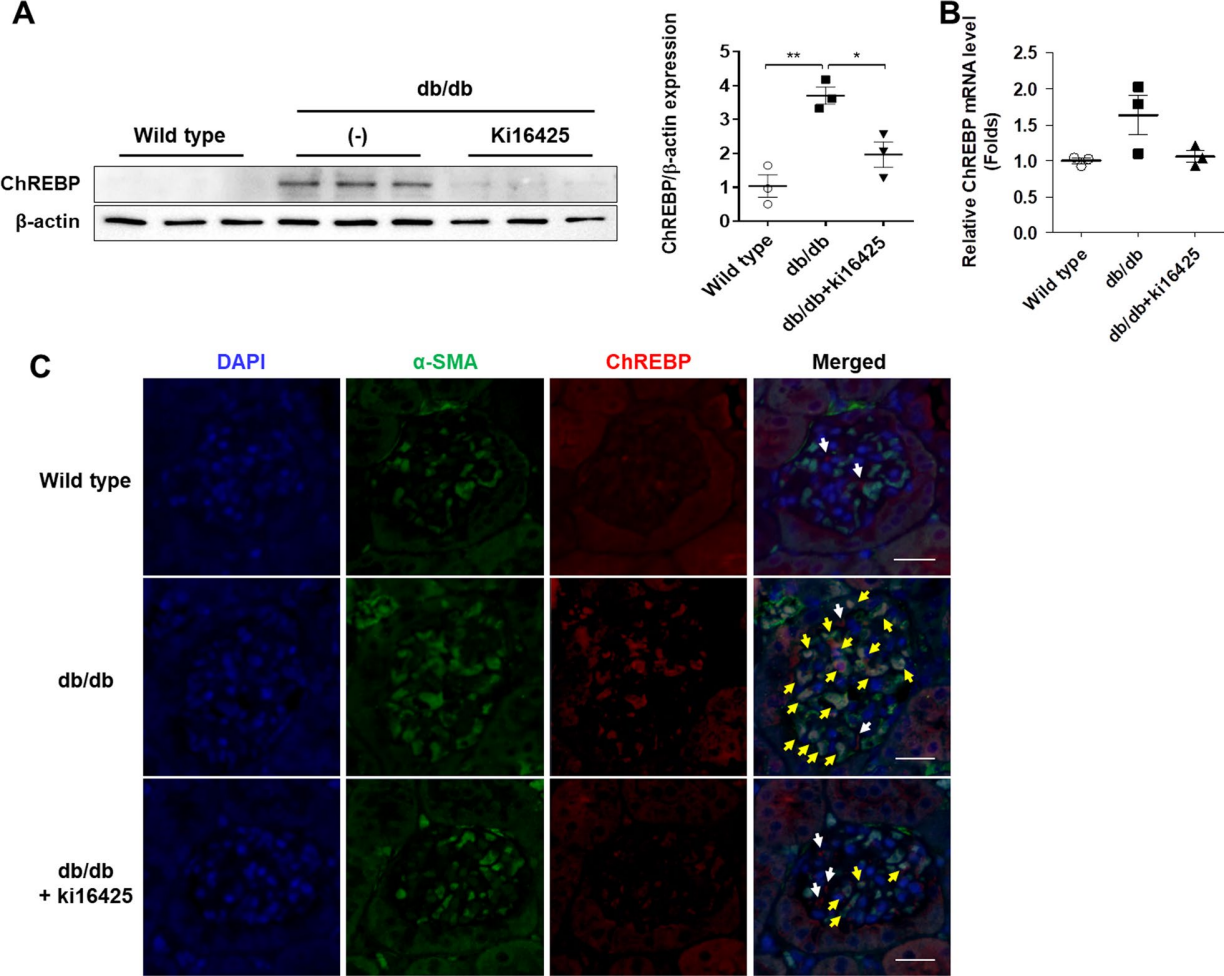
ChREBP expression is reduced in the renal cortex of db/db mice treated with the LPAR inhibitor, ki16425. Eight-week-old wild-type and db/db mice were intraperitoneally injected with the vehicle or ki16425 (10 mg/kg) for 8 weeks on a daily basis, following which the mice were sacrificed. **A** The level of ChREBP protein in the renal cortex was analyzed by western blotting, quantified using ImageJ software, and normalized to those of β-actin. **B** The level of ChREBP mRNA in the renal cortex was determined by quantitative real-time polymerase chain reaction (qRT-PCR). The data are presented as the mean ± SEM (n = 3/group); *p < 0.05, **p < 0.01. **C** Representative images depicting the colocalization of α-SMA (green) and ChREBP (red) in the kidney tissue sections of mice. The nuclei were counterstained with DAPI (blue). The white arrows indicate the cytosolic expression of ChREBP, and the yellow arrows indicate the nuclear expression of ChREBP in α-SMA-positive cells. Scale bars, 20 μm; n = 3

We have previously reported that LPA induces the fibrosis of renal mesangial cells, and that treatment with ki16425, an LPAR1/3 antagonist, inhibits renal fibrosis and improves DN in db/db type 2 diabetic mice [10]. In order to determine the effect of ki16425 treatment on the renal expression of ChREBP in db/db mice, we measured the expression level of ChREBP protein and mRNA in the renal cortex by western blotting and qRT-PCR, respectively. The expression of ChREBP protein significantly increased in the kidney tissues of db/db mice compared to that in the wild-type mice; however, the increase in the expression of ChREBP protein was significantly suppressed by the administration of ki16425 (Fig. 1A). Although the expression of ChREBP mRNA increased in the renal cortex of db/db diabetic mice compared to that of the wild-type mice, the difference was not significant (Fig. 1B). In addition, the administration of ki16425 in db/db mice led to maintaining the expression level of ChREBP mRNA similar to that in wild-type mice (Fig. 1B). In order to identify the cell types expressing ChREBP in the kidney, the kidney sections were subjected to IFA staining. The expression of ChREBP protein increased significantly in the glomerulus, particularly in cells expressing α-SMA, a mesangial cell marker [20–22], and was inhibited by the administration of ki16425 in db/db mice. Although most of the glomeruli in wild-type mice were negative for α-SMA expression (Additional file 1: Fig. S1), some mesangial cells (about 5.6% of the total number of glomeruli) expressed α-SMA in wild-type mice (Fig. 1C). ChREBP expression in wild-type mice was observed in cytoplasm of a very small number of α-SMA positive cells, but not in the nuclei (Fig. 1C). However, nuclear expression of ChREBP in mesangial cells was significantly increased in db/db mice and was inhibited by the administration of ki16425 (Fig. 1C). We did not observe any changes in the expression of ChREBP protein in the renal tubules of 16-week-old db/db mice, although it has been reported that the expression of ChREBP increases in the renal glomeruli and tubules of patients with DN and animal models of diabetes [23]. However, these observations could not be confirmed by co-staining with podocyte markers. These results indicated that the expression of ChREBP protein primarily increased in glomerular mesangial cells of the renal cortex, suggesting the involvement of ChREBP in LPA-mediated renal fibrosis and progression of DN.

### LPA induced the expression of ChREBP and fibrotic factors in SV40 MES13 cells

**Fig. 2:**
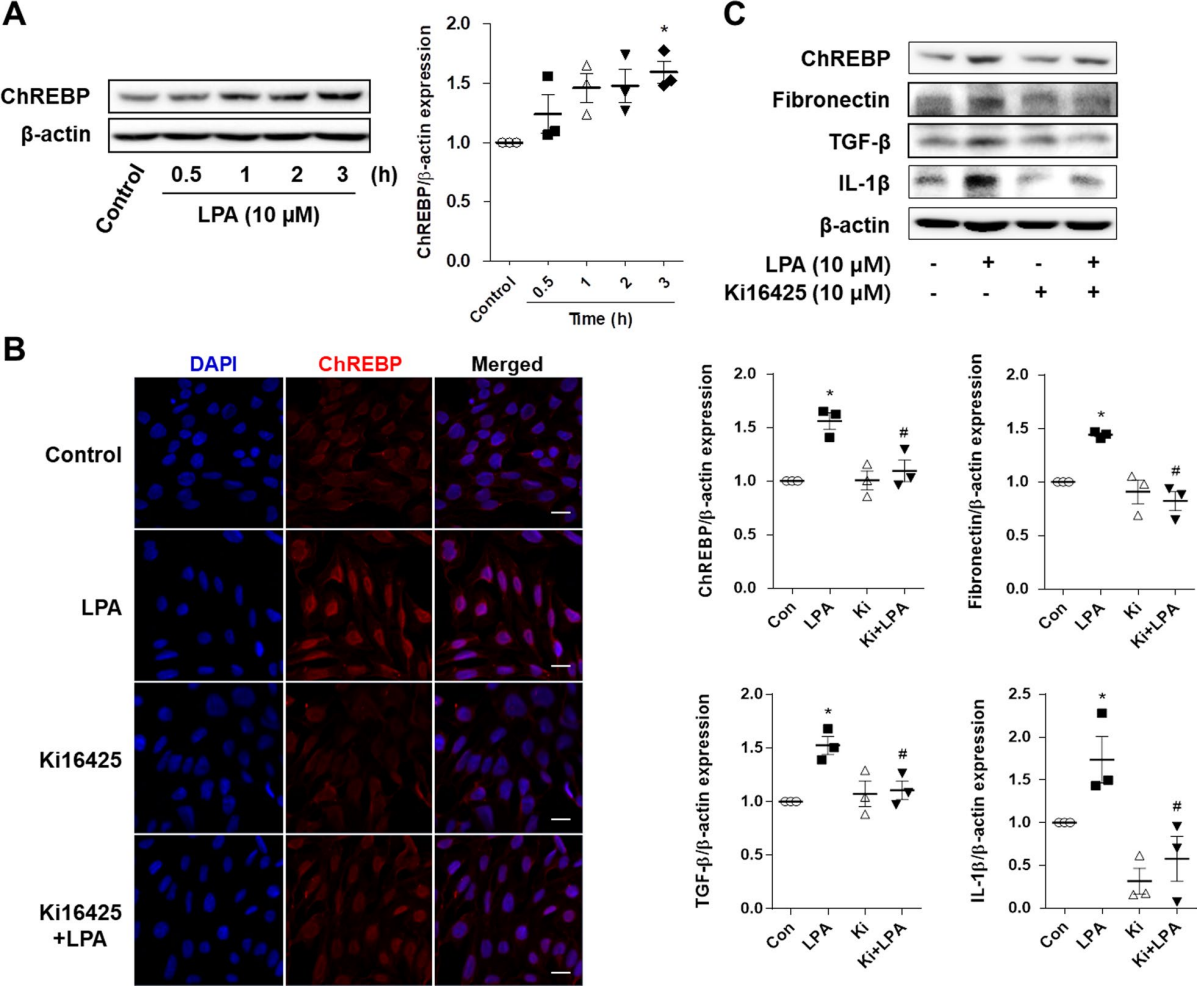
LPA induces the expression of ChREBP and fibrotic factors in SV40 MES13 cells. **A** SV40 MES13 cells were treated with 10 µM LPA for the indicated durations. The level of ChREBP protein was analyzed by western blotting, quantified using ImageJ software, and normalized to that of β-actin. The data are presented as the mean ± SEM of results obtained from three independent experiments. *p < 0.05 vs. control. **B** SV40 MES13 cells were seeded in 4-well chambers and treated with LPA in the presence or absence of ki16425 for 3 h. Immunocytochemistry analysis was performed using an anti-ChREBP antibody and an Alexa Fluor 546-conjugated secondary antibody. The nuclei were counterstained with DAPI (blue) (original magnification, 400 ×; the scale bars represent 20 μm). **C** The SV40 MES13 cells were treated with LPA in the presence or absence of ki16425 for 3 h. The protein levels of ChREBP, fibronectin, TGF-β, and IL-1β were analyzed by western blotting, quantified using ImageJ software, and normalized to those of β-actin. The data are presented as the mean ± SEM of results obtained from three independent experiments. *p < 0.05 vs. control (Con); ^#^p < 0.05 vs. LPA

In order to determine whether LPA induces the expression of ChREBP and fibrotic factors in renal mesangial cells, we examined the expression of ChREBP, fibronectin, TGF-β, and IL-1β in SV40 MES13 cells treated with LPA. In a previous report [10], we observed that LPA significantly increased the expression of TGF-β1, a major fibrotic factor, in SV40 MES13 cells treated with 10 µM LPA for 3 h. In this study, the experiments were therefore performed with LPA at a concentration of 10 μM, and the expression of ChREBP was analyzed in SV40 MES13 cells following treatment with LPA. The expression of ChREBP protein tended to increase from 1 h and significantly increased after 3 h of treatment with LPA, compared to that in the control cells (Fig. 2A). As depicted in Fig. 2B, the expression of ChREBP, especially in the nucleus, was greatly increased in the LPA-treated SV40 MES13 cells compared to that in the control cells, however, this increased fluorescence intensity was decreased by treatment with ki16425 (Fig. 2B). We also confirmed this result at the protein level by Western blotting analysis (Fig. 2C). Treatment with LPA significantly increased the protein expression of fibrotic factors, including fibronectin, TGF-β, and IL-1β, compared to that in the control cells (Fig. 2C). However, treatment with ki16425, significantly suppressed the expression of these fibrotic factors, compared to that in the LPA-treated cells (Fig. 2C). These results indicated that LPA induced the expression of ChREBP and fibrotic factors, and their expression was positively correlated in SV40 MES13 cells.

### ChREBP regulated the LPA-induced expression of fibrotic factors in SV40 MES13 cells

**Fig. 3:**
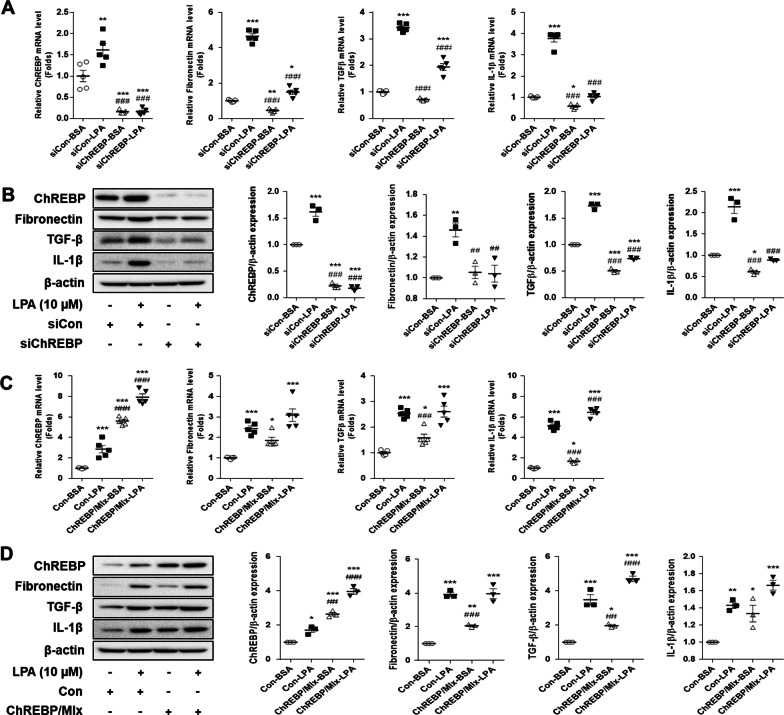
ChREBP regulates the LPA-induced expression of fibrotic factors in SV40 MES13 cells. **A** and **B** SV40 MES13 cells were transfected with a control siRNA (siCon) or ChREBP siRNA (siChREBP), and treated with 10 µM LPA for 3 h. The mRNA (A) and protein (**B**) levels of ChREBP, fibronectin, TGF-β, and IL-1β were analyzed by qRT-PCR (n = 5) and western blotting (n = 3), respectively. **C** and **D** The SV40 MES13 cells were co-transfected with vectors expressing ChREBP and Mlx (ChREBP/Mlx), or transfected with an empty pcDNA vector (Con), and treated with 10 µM LPA for 3 h. The mRNA (**C**) and protein (**D**) levels of ChREBP, fibronectin, TGF-β, and IL-1β were analyzed by qRT-PCR (n = 5) and western blotting (n = 3), respectively. The data are presented as the mean ± SEM. *p < 0.05, **p < 0.01, ***p < 0.005 vs. siCon-BSA or Con-BSA; ^#^p < 0.05, ^##^p < 0.01, ^###^p < 0.005 vs. siCon-LPA or Con-LPA

In order to investigate the role of ChREBP in the fibrotic response, the cells were transfected with ChREBP siRNA or the overexpression vector system, and the expression of the fibrotic factors in the LPA-treated cells was measured (Fig. 3). The expression levels of ChREBP mRNA and protein significantly decreased following transfection with ChREBP siRNA. The LPA-induced upregulation of fibrotic factors, including fibronectin, TGF-β, and IL-1β was also significantly reduced by ChREBP knockdown, compared to that in the cells transfected with control siRNA (Fig. 3A and B). The expression levels of ChREBP mRNA and protein significantly increased following transfection with ChREBP/Mlx vectors compared to those in the cells transfected with the control vector (Fig. 3C and D). The overexpression of ChREBP significantly increased the mRNA and protein expression of the fibrotic factors compared to that in the control group. The ChREBP-induced mRNA and protein expression of the fibrotic factors was further enhanced when the cells overexpressing ChREBP were treated with LPA (Fig. 3C and D). These results suggested that ChREBP regulated the LPA-induced expression of fibrotic factors in SV40 MES13 cells.

### LPA induced ROS production, and ROS scavenging decreased LPA- or ROS-induced expression of ChREBP and fibrotic factors in SV40 MES13 cells

**Fig. 4:**
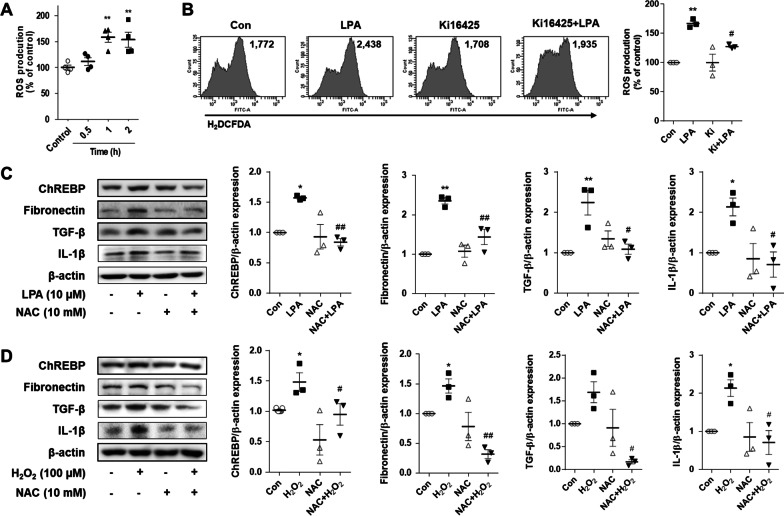
LPA induces the production of ROS, and ROS scavenging decreases the LPA- or ROS-induced expression of ChREBP and fibrotic factors in SV40 MES13 cells. **A** SV40 MES13 cells were treated with 10 µM LPA for the indicated durations. **B** SV40 MES13 cells were treated with LPA in the presence or absence of ki16425 for 1 h. **A** and **B** The intracellular levels of ROS were measured by flow cytometry with DCFH-DA. The values in the representative flow cytometry histograms indicate the fluorescence intensity of DCF in the whole cells. The relative fluorescence intensity levels of DCF were compared with the production of ROS in the control cells. **C** The cells were pretreated with NAC for 1 h and subsequently treated with LPA for 3 h. **D** The cells were pretreated with NAC for 1 h and subsequently treated with H_2_O_2_ for 1 h. **C** and **D** The protein levels of ChREBP, fibronectin, TGF-β, and IL-1β were analyzed by western blotting, quantified using ImageJ software, and normalized to those of β-actin. The data are presented as the mean ± SEM of results obtained from three independent experiments. *p < 0.05, **p < 0.01 vs. Con; ^#^p < 0.05, ^##^p < 0.01 vs. LPA or H_2_O_2_

Previous studies have demonstrated that LPA induces the generation of ROS in various cells [24–26], and the increased levels of ROS promote the expression of ChREBP in liver cells [15]. We therefore investigated whether the LPA-induced expression of ChREBP and fibrotic factors was mediated via ROS production in SV40 MES13 cells. We first measured the changes in the intracellular levels of ROS induced by LPA signaling in SV40 MES13 cells by flow cytometry. The intracellular production of ROS increased significantly after 1 h of treatment with LPA compared to that in the control cells (Fig. 4A). The LPA-induced production of ROS was significantly reduced by co-treatment with ki16425 compared to that in the LPA-treated cells (Fig. 4B). In order to confirm whether the LPA-induced production of ROS mediates the expression of ChREBP and fibrotic factors, the SV40 MES13 cells were pretreated with NAC, an ROS scavenging agent, and subsequently treated with LPA. As depicted in Fig. 4C, pretreatment with NAC significantly reduced the expression of ChREBP and fibrotic factors, including fibronectin, TGF-β, and IL-1β, compared to that of the cells exposed to LPA alone (Fig. 4C). Consistently, the expression of ChREBP and fibrotic factors significantly increased in the cells treated with H_2_O_2_, compared to that in the control cells. The H_2_O_2_-induced expression of ChREBP and fibrotic factors was suppressed by pretreatment with NAC (Fig. 4D). These results indicated that the LPA-induced expression of ChREBP and fibrotic factors was mediated by ROS in renal mesangial cells.

### LPA induced the expression of ChREBP and fibrotic factors by downregulating Smurf2 in SV40 MES13 cells

**Fig. 5:**
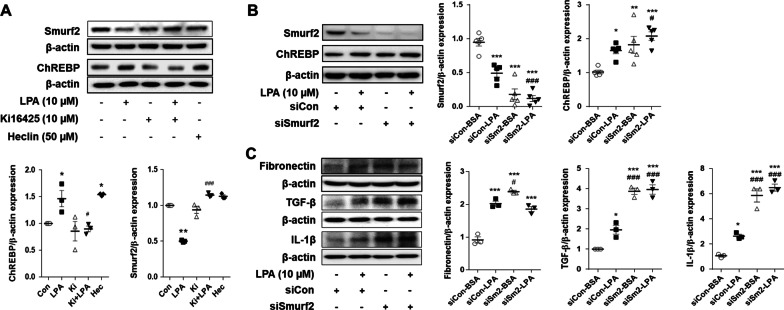
LPA induces the expression of ChREBP and fibrotic factors via Smurf2 in SV40 MES13 cells. **A** SV40 MES13 cells were treated with LPA, either in the presence or absence of ki16425, or treated with Heclin (Hec) for 3 h. **B** and **C** The SV40 MES13 cells were transfected with a control siRNA (siCon) or Smurf2 siRNA (siSmurf2, siSm2) for 6 h, then the medium was replaced with SFM for 16–18 h, and treated with LPA for 3 h. The proteins levels of ChREBP, Smurf2, fibronectin, TGF-β, and IL-1β were analyzed by western blotting, quantified using ImageJ software, and normalized to those of β-actin. The data are presented as the mean ± SEM of results obtained from three to five independent experiments. *p < 0.05, **p < 0.01 vs. Con or siCon-BSA; ^#^p < 0.05, ^##^p < 0.01, ^###^p < 0.005 vs. LPA or siCon-LPA

We observed that LPA significantly increased the mRNA and protein levels of ChREBP in SV40 MES13 cells (Fig. 3). In order to investigate the effect of transcription on the LPA-induced expression of ChREBP, SV40 MES13 cells were pretreated with actinomycin D, a transcriptional inhibitor, and subsequently treated with LPA. The LPA-induced expression of ChREBP protein was significantly inhibited by actinomycin D, but was decreased by only 30% compared to that in the LPA-treated cells, and was still significantly higher than that in the control group (Additional file 1: Fig. S2). We therefore investigated the mechanisms underlying the stabilization of the ChREBP protein in LPA-treated SV40 MES13 cells. As the protein levels of ChREBP are regulated by the E3 ubiquitin ligase, Smurf2 [27], we investigated whether Smurf2 is involved in the fibrotic response in LPA-treated SV40 MES13 cells. LPA decreased the expression of Smurf2 compared to that in the control cells; however, treatment with ki16425 significantly restored the LPA-induced reduction in Smurf2 expression (Fig. 5A). To inhibit Smurf2 activity, Heclin, an inhibitor of HECT domain E3 ubiquitin ligase [28], was used. Heclin inhibits the formation of a Ub-E3 thioester bond by inducing conformational changes that induce the spontaneous oxidation of active site cysteines [28]. Treatment with Heclin significantly increased the expression of ChREBP in SV40 MES13 cells, similar to that of the cells that were only treated with LPA (Fig. 5A). In order to confirm that Smurf2 regulates the LPA-induced expression of ChREBP and fibrotic factors, SV40 MES13 cells were transfected with Smurf2 siRNA and subsequently treated with LPA. The expression of Smurf2 was significantly reduced in the cells transfected with Smurf2 siRNA, compared to that of the cells transfected with the control siRNA, and was further reduced following LPA treatment (Fig. 5B). Conversely, the expression of ChREBP was significantly increased by Smurf2 knockdown, similar to that of the LPA-treated cells that were transfected with the control siRNA, and was further increased by LPA treatment (Fig. 5B). Consistent with the changes in the expression of ChREBP, the expression of the fibrotic factors, including fibronectin, TGF-β, and IL-1β, was also significantly increased by Smurf2 knockdown, compared to that of the cells transfected with the control siRNA (Fig. 5C). These results indicated that Smurf2 regulated the LPA-induced expression of ChREBP and fibrotic factors in renal mesangial cells.

### The LPA-induced expression of ChREBP was mediated via the inhibition of ChREBP ubiquitination by the downregulation of Smurf2 in SV40 MES13 cells

**Fig. 6:**
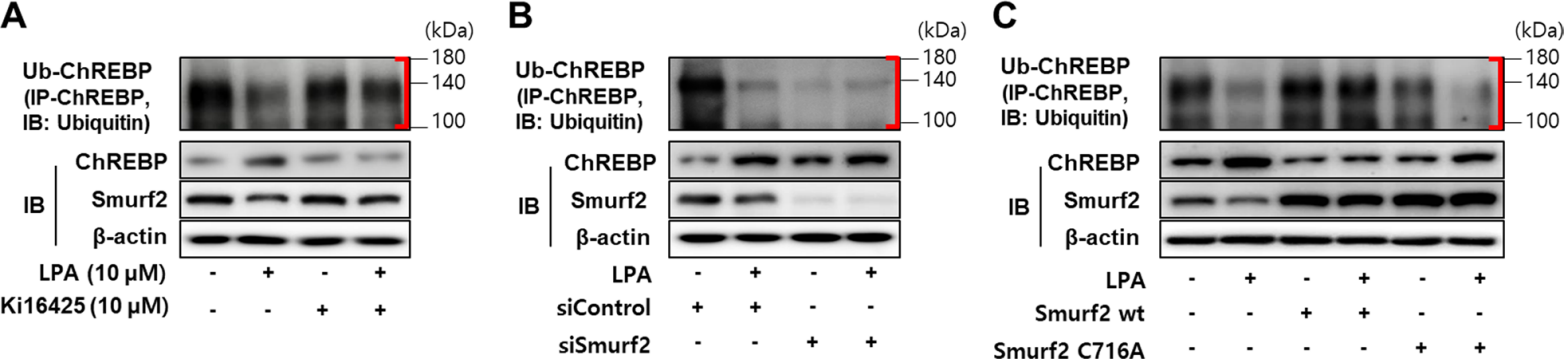
LPA inhibits the ubiquitination of ChREBP by downregulating Smurf2 in SV40 MES13 cells. **A** SV40 MES13 cells were treated with LPA in the presence or absence of ki16425 for 3 h. **B** The cells were transfected with a control siRNA (siCon) or Smurf2 siRNA (siSmurf2) for 6 h, then the medium was replaced with SFM for 16–18 h, and subsequently treated with LPA for 3 h. **C** The cells were transfected with pCMV5B-Flag-Smurf2 wt (Smurf2 wt) or pCMV5B-Flag-Smurf2 C716A (Smurf2 C716A) for 6 h, then the medium was replaced with SFM for 16–18 h, and subsequently treated with LPA for 3 h. (A-C) The cell lysates were subjected to immunoprecipitation studies by incubating with an anti-ChREBP antibody followed by blotting with anti-ubiquitin antibody. The protein levels of ubiquitinated-ChREBP (Ub-ChREBP), ChREBP, and Smurf2 were analyzed by western blotting. Representative images of the blots from three independent experiments are depicted, where the red brackets indicate Ub-ChREBP

In order to investigate whether the LPA-induced downregulation of Smurf2 actually increases the expression of ChREBP by inhibiting the ubiquitin-mediated degradation of ChREBP, we analyzed the expression of ubiquitinated ChREBP in LPA-treated SV40 MES13 cells. The expression of ubiquitinated ChREBP significantly reduced in the LPA-treated cells, compared to that of the control cells; however, co-treatment with ki16425 restored the expression of ubiquitinated ChREBP compared to that in the cells that were only treated with LPA (Fig. 6A). Moreover, the knockdown of Smurf2 significantly reduced the ubiquitination of ChREBP in cells with or without LPA treatment, compared to that in the cells transfected with the control siRNA (Fig. 6B). The overexpression of wild-type Smurf2 significantly increased the expression of ubiquitinated ChREBP compared to that in the control cells or the cells transfected with the catalytically inactive Smurf2 with a C716A mutation (Fig. 6C). These results suggested that LPA decreased the ubiquitination and degradation of ChREBP by downregulating Smurf2, thereby increasing the expression of ChREBP and inducing the fibrotic response.

### LPA decreased the expression of Smurf2 via Akt phosphorylation, and subsequently increased the expression of ChREBP in SV40 MES13 cells

**Fig. 7:**
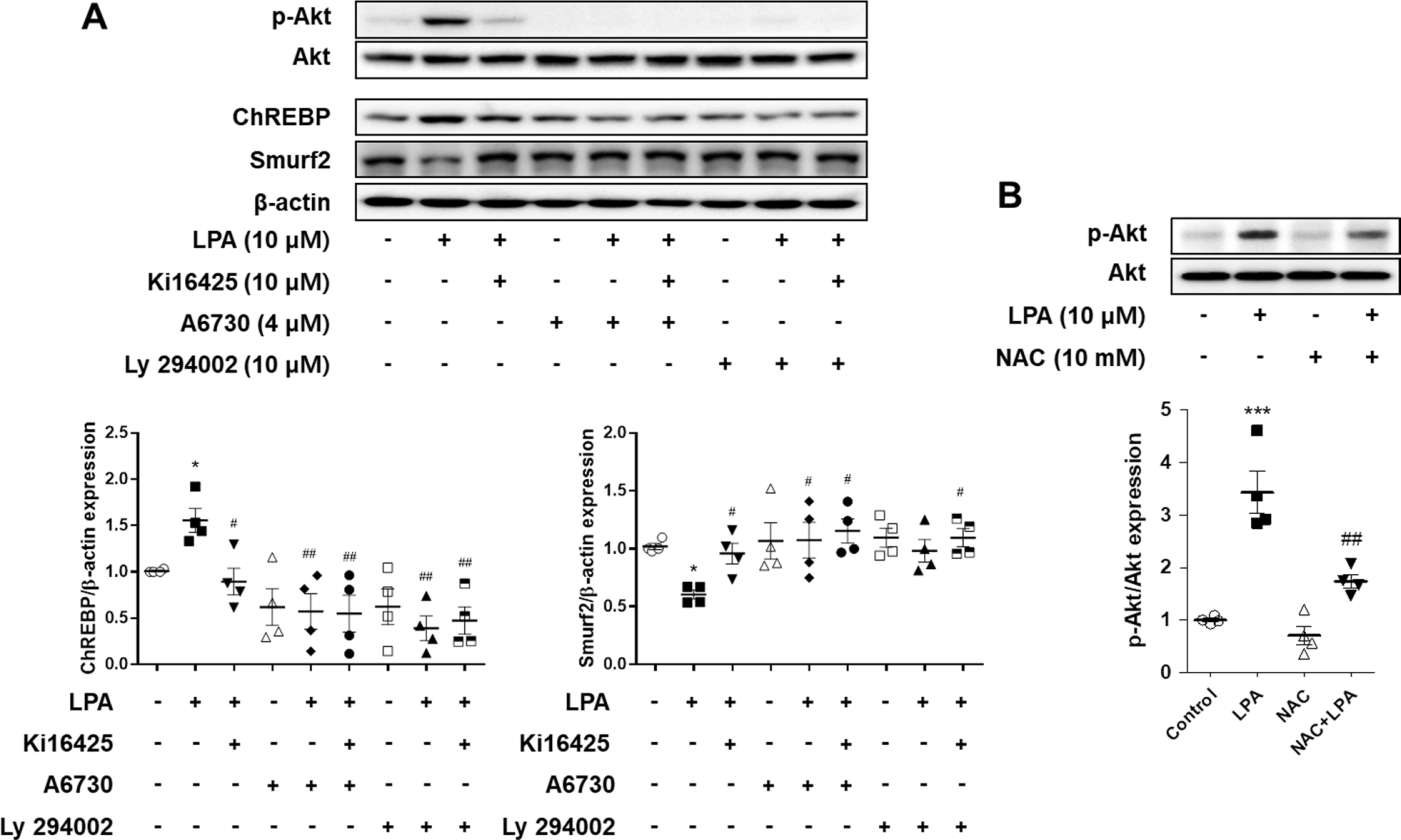
Inhibition of Akt signaling suppresses the alterations in the expression of ChREBP and Smurf2 induced by LPA. **A** SV40 MES13 cells were pretreated with A6730 (Akt1/2 kinase inhibitor, 4 μM) or Ly 294002 (PI3K inhibitor, 10 μM) for 1 h, and subsequently treated with LPA and/or ki16425 for 3 h. **B** SV40 MES13 cells were pretreated with NAC for 1 h and subsequently treated with LPA for 3 h. **A** and **B** The protein levels of p-Akt, ChREBP, and Smurf2 were analyzed by western blotting, quantified using ImageJ software, and normalized to those of β-actin. The data are presented as the mean ± SEM of results obtained from four independent experiments. *p < 0.05, **p < 0.01 vs. vehicle only (control); ^#^p < 0.05, ^##^p < 0.01 vs. LPA only

It is known that Akt, a serine/threonine kinase, is an upstream inhibitor of Smurf2 [27] and LPA induces the phosphorylation of Akt [26]. We therefore determined the expression of p-Akt and Smurf2 in LPA-treated SV40 MES13 cells in this study. The results demonstrated that the expression of p-Akt significantly increased, while the expression of Smurf2 significantly decreased, following LPA treatment. The phosphorylation of Akt was significantly inhibited by treatment with ki16425 or pretreatment with a pharmacological inhibitor of Akt (Fig. 7A). In parallel, pretreatment with an Akt inhibitor or PI3K inhibitor restored the LPA-induced reduction in the expression of Smurf2, and significantly reduced the LPA-induced expression of ChREBP (Fig. 7A). In order to determine whether the LPA-induced phosphorylation of Akt is inhibited by ROS scavenging, SV40 MES13 cells were pretreated with NAC and subsequently treated with LPA. The results demonstrated that LPA increased the phosphorylation of Akt compared to that in the control cells, and that pretreatment with NAC significantly inhibited the LPA-induced phosphorylation of Akt (Fig. 7B). These results suggest that the LPA-induced production of ROS mediates Akt phosphorylation in mesangial cells, which induces the downregulation of Smurf2 and increased expression of ChREBP, leading to a fibrotic response.

### LPA increased Traf4-mediated ubiquitination of Smurf2 in SV40 MES13 cells

**Fig. 8:**
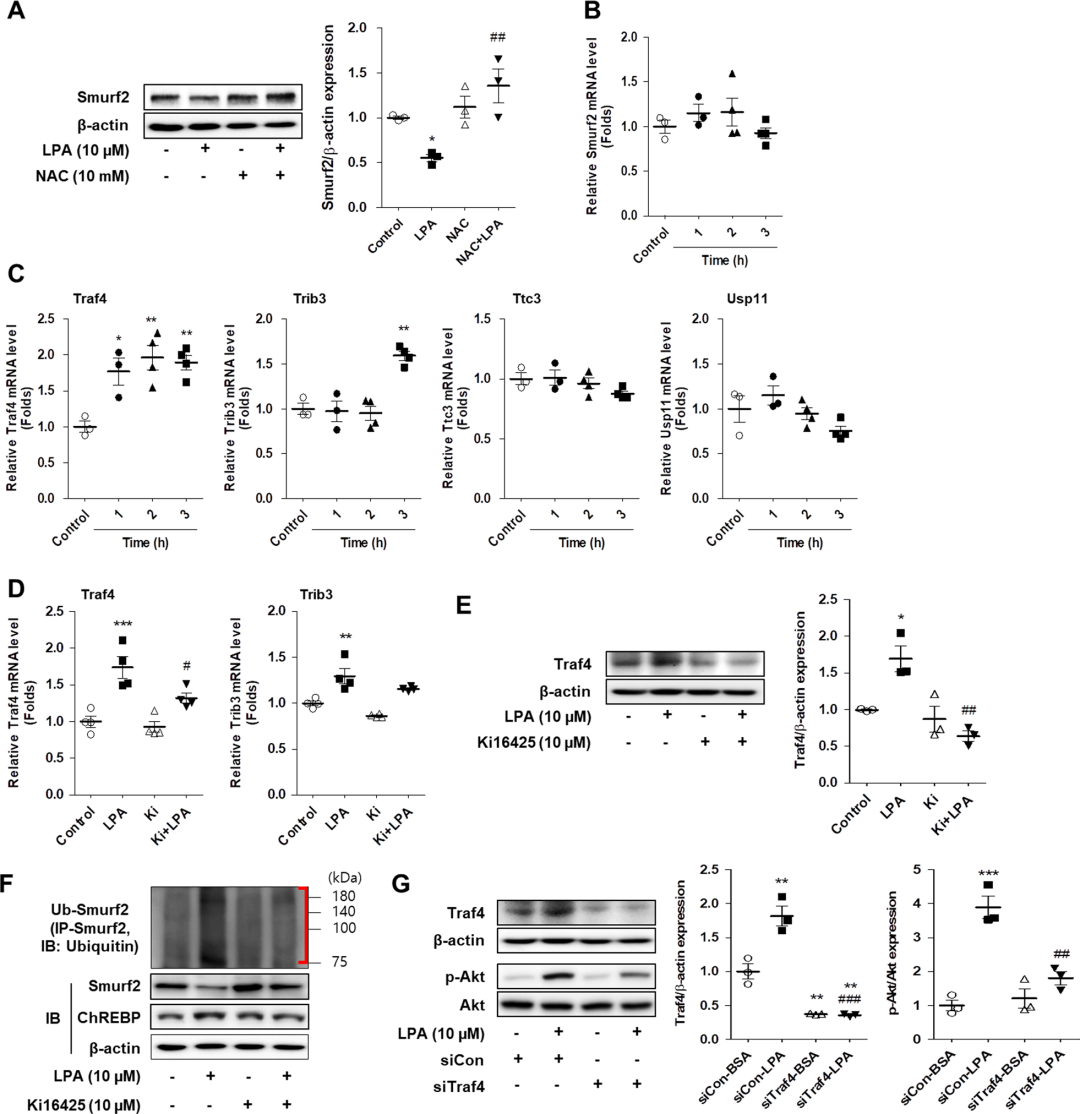
LPA increases Traf4-mediated ubiquitination of Smurf2 in SV40 MES13 cells. **A** SV40 MES13 cells were pretreated with NAC for 1 h and subsequently treated with LPA for 3 h. The level of Smurf2 protein was analyzed by western blotting, quantified using ImageJ software, and normalized to that of β-actin. **B** and **C** SV40 MES13 cells were treated with 10 µM LPA for the indicated durations. The mRNA levels of Smurf2 (**B**) and ubiquitin ligases (**C**), including Traf4, Trib3, Ttc3, and USP11, were determined by qRT-PCR. **D**–**F** SV40 MES13 cells were treated with LPA in the presence or absence of ki16425 for 3 h. **D** The mRNA levels of Traf4 and Trib3 were determined by qRT-PCR. **E** The level of Traf4 protein was analyzed by western blotting, quantified using ImageJ software, and normalized to that of β-actin. **F** The cell lysates were subjected to immunoprecipitation studies by incubating with an anti-Smurf2 antibody followed by blotting with anti-ubiquitin antibody. The protein levels of ubiquitinated-Smurf2 (Ub-Smurf2), ChREBP, and Smurf2 were analyzed by western blotting. Representative images of the blots from three independent experiments are depicted, where the red brackets indicate Ub-Smurf2. **G** SV40 MES13 cells were transfected with a control (siCon) or Traf4 (siTraf4) siRNA for 6 h, then the medium was replaced with SFM for 16–18 h, and treated with LPA for 3 h. The proteins levels of Traf4 and p-Akt were analyzed by western blotting, quantified using ImageJ software, and normalized to those of β-actin and Akt, respectively. The data are presented as the mean ± SEM of results obtained from four independent experiments. *p < 0.05, **p < 0.01 vs. vehicle only (control) or siCon-BSA; ^#^p < 0.05, ^##^p < 0.01 vs. LPA only or siCon-LPA

In order to investigate the effect of ROS scavenging on the expression of Smurf2, SV40 MES13 cells were pretreated with NAC and subsequently treated with LPA. LPA-induced downregulation of Smurf2 was restored by pretreatment with NAC (Fig. 8A), indicating that the expression of Smurf2 was reduced by ROS generated during treatment with LPA. We next analyzed the expression of Smurf2 mRNA following LPA treatment to investigate whether LPA repressed the transcription of Smurf2. LPA did not alter the expression of Smurf2 mRNA until 3 h (Fig. 8B), suggesting that the stability of the Smurf2 protein was reduced by LPA. It has been reported that the downregulation of Smurf2 via the phosphorylation of Akt is attributed to the increased ubiquitination of Smurf2 [27, 29], and that the ubiquitination of Smurf2 is regulated by RING domain E3 ubiquitin ligases, including Traf4, Trib3, Ttc3, and Usp11 [30]. We therefore examined the mRNA expression of Traf4, Trib3, Ttc3, and Usp11 in SV40 MES13 cells following treatment with LPA. LPA significantly increased the expression of Traf4 and Trib3 mRNA, but not that of Ttc3 and Usp11 mRNA (Fig. 8C). Treatment with ki16425 significantly inhibited the LPA-induced expression of Traf4 mRNA, but not Trib3 mRNA (Fig. 8D). In addition, LPA significantly increased Traf4 protein expression compared to that of control cells and ki16425 significantly inhibited the LPA-induced expression of Traf4 (Fig. 8E). Consistent with these results, LPA increased the ubiquitination of Smurf2, while ki16425 inhibited LPA-induced Smurf2 ubiquitination (Fig. 8F). We then investigated whether LPA-induced phosphorylation of Akt is regulated by Traf4 and found that knockdown of Traf4 using Traf4 siRNA significantly reduced LPA-induced Akt phosphorylation compared to control siRNA-transfected cells (Fig. 8G). These results suggest that LPA increases the ubiquitination of Smurf2 by upregulating the expression of RING domain E3 ligases, mainly Traf4, in SV40 MES13 cells.

### Smurf2 expression was restored in the glomeruli of db/db mice treated with ki16425

**Fig. 9:**
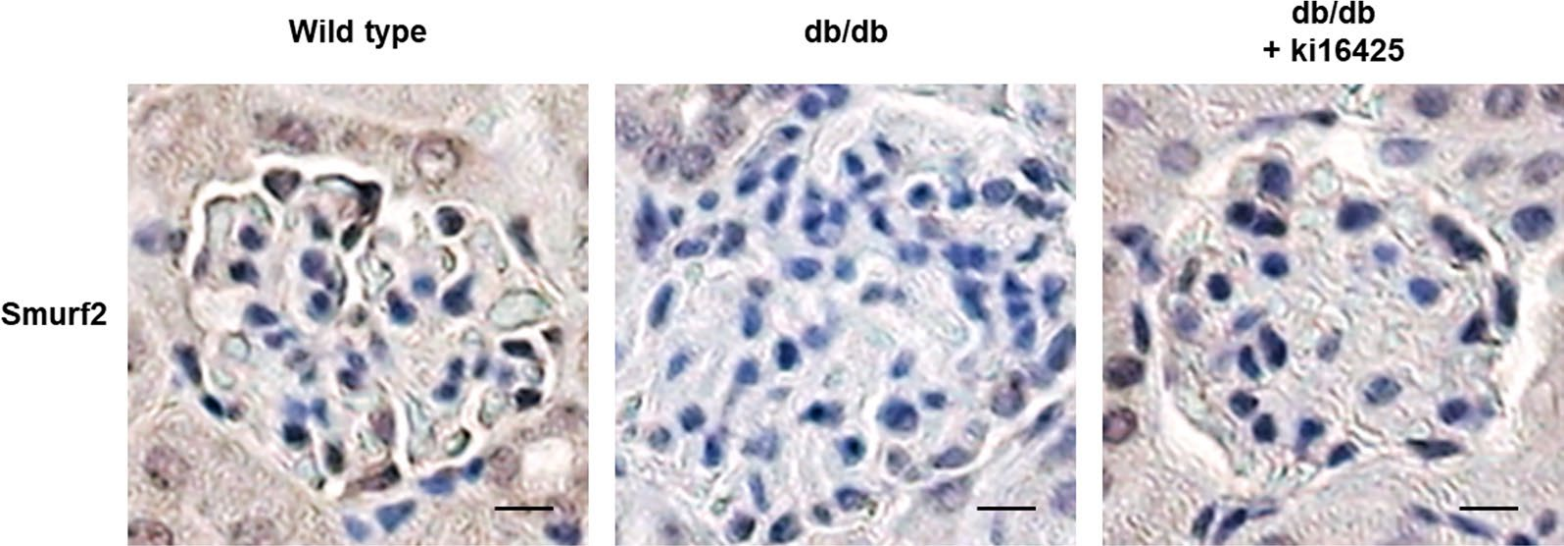
Smurf2 expression is restored in the glomeruli of db/db mice treated with the ki16425. Eight-week-old wild-type and db/db mice were intraperitoneally injected with the vehicle or ki16425 (10 mg/kg/day) for 8 weeks and the mice were sacrificed. Immunohistochemical detection of Smurf2 was performed in the kidney tissues using DAB. The nuclei were counterstained with hematoxylin. The representative images are depicted (original magnification, 200 ×; scale bars, 20 μm; n = 3)

To confirm the effect of ki16425 treatment on the renal expression of Smurf2 in db/db mice, we analyzed the expression levels of these molecules in kidney tissue sections by immunohistochemical staining. Consistent with the results in mesangial cells, the glomerular expression of Smurf2 remarkably decreased in db/db mice compared to that in the wild-type mice; however, the decrease in Smurf2 expression was significantly suppressed by the administration of ki16425 (Fig. 9). These results confirm that LPA-induced decrease in Smurf2 expression is involved in the progression of diabetic nephropathy not only in mesangial cells in vitro but also in glomerular tissues in vivo.

## Discussion

DN, a major complication of diabetes, involves glomerulosclerosis and fibrosis caused by metabolic and hemodynamic changes in the kidneys, resulting from diabetes. Although DN contributes to overall morbidity and mortality in diabetic patients, the pathophysiological mechanism of DN remains poorly understood [31, 32]. A multidisciplinary therapeutic strategy is generally employed for DN, including stringent glucose control, blood pressure control with renin-angiotensin system (RAS) inhibitors, and the use of sodium-glucose co-transporter 2 (SGLT2) inhibitors. Despite the multi-therapeutic approach, it is not feasible to completely suppress the progression of DN [32].

Renal fibrosis is the final pathological outcome of most chronic progressive kidney diseases, including DN [4]. The pathological features of the glomeruli during the progression of DN include mesangial expansion, thickening of the basement membrane, loss of podocytes, and glomerulosclerosis [3, 33]. Several studies, including our previous report, have demonstrated that LPA induces the hyperproliferation of mesangial cells [7, 34–36], and that the pharmacological blockade of LPA/LPAR signaling inhibits renal fibrosis and improves DN in diabetic mice [10, 11, 37]. Consistent with these reports, it has been demonstrated that the levels of LPA are significantly increased in the glomeruli of diabetic mice [38, 39] and high-fat diet-induced obese mice [40]. In addition, a previous study reported that the levels of LPA and precursor lysophosphatidyl choline (LPC) in the urine are significantly higher in diabetic patients with DN than in diabetic patients without DN [41]. Furthermore, urinary levels of LPC increase in patients with diabetic nephropathy showing rapidly progressing kidney dysfunction, which was correlated with a decline in estimated glomerular filtration rate after 2.5 years of the basal assessment [42]. These findings suggest that LPA might be a crucial factor for the development of renal fibrosis in DN. However, the molecular mechanisms underlying the fibrotic response in mesangial cells during the progression of DN remain unclear.

ChREBP, a carbohydrate sensing transcription factor, has been primarily studied for its regulatory effects on the glycolytic and lipogenesis pathways in hepatic and adipose tissues [43]. Despite the scarcity of studies on the function of ChREBP in the kidneys, it is known that the renal expression of ChREBP increases in rats with chronic kidney disease [44], in Akita type 1 diabetic mice [45], and in mice and rats with STZ-induced diabetes [16, 46]. Another recent study demonstrated that the expression of ChREBP protein increases significantly in the renal glomeruli and tubules of patients with DN [23]. Additionally, the ablation of ChREBP reduces the symptoms of DN, including glomerular hypertrophy, in mice with STZ-induced diabetes [46], suggesting that ChREBP may play an important role in the progression of DN. We therefore aimed to investigate whether ChREBP is involved in the LPA-mediated renal fibrosis in type 2 diabetic mice. To this end, we initially determined the expression of ChREBP in the renal cortex of db/db mice treated with the LPAR1/3 antagonist, ki16425. We observed that the expression of ChREBP protein primarily increased in the glomerular mesangial cells of the renal cortex in the db/db mice, but reduced in the db/db mice treated with ki16425. This indicated that the expression ChREBP could play a role in the LPA-mediated renal fibrosis in diabetic mice. In vitro studies investigating the suppression or overexpression of ChREBP confirmed that ChREBP regulates the LPA-induced expression of fibrotic factors in SV40 MES13 cells. Some experiments revealed an additional LPA response even after transfection with the overexpression vectors. This might be due to the low transfection efficiency, which was about 30% (Additional file 1: Fig. S3). We have previously reported that LPA promotes cellular proliferation via the Rac1/MAPK/KLF5 signaling pathway in SV40 MES13 mesangial cells [7]. The basal cell growth was not altered irrespective of ChREBP knockdown (Additional file 1: Fig. S4A) or overexpression (Additional file 1: Fig. S4B). However, LPA-induced cellular proliferation decreased significantly following ChREBP knockdown at both 24 and 48 h, and increased significantly following ChREBP overexpression. Although the relationship between ChREBP and the Rac1/MAPK/KLF5 signaling pathway remains to be investigated, these results indicate that ChREBP is involved in the LPA-induced proliferation of SV40 MES13 cells.

ROS act as important physiological regulators of intracellular signaling pathways [47], and it has been demonstrated that LPA induces the production of ROS in a variety of cell types, including mesangial cells [11, 24–26]. Among the ROS-producing enzymes, NADPH oxidase 4 (Nox4) is expressed at highest levels in the kidney and is a major contributor to the production of ROS in the diabetic kidney [48, 49]. It has been reported that high levels of glucose induce the Nox4-dependent production of ROS in mesangial cells [50, 51]. Additionally, we have previously reported that LPA induces the Nox-dependent production of ROS in SV40 MES13 cells [11]. Chen et al. also observed that the AGEs-induced production of ROS promotes the expression of ChREBP in liver cancer cells [15]. We therefore investigated whether the LPA-induced production of ROS affects the expression of ChREBP. The results of this study demonstrated that the production of ROS by LPA or exogenous treatment with H_2_O_2_ increased the expression of ChREBP and fibrotic factors in mesangial cells. Similar to our results, previous studies have reported that the production of ROS by high levels of glucose induces fibrotic injury in mesangial cells [50, 52]. Conversely, it has been also reported that the endogenous ChREBP activated by high levels of glucose induces the production of ROS in renal proximal tubular cells [53], implying that ROS and ChREBP can possibly influence each other depending on the cell type and stimulator. In this study, ROS scavenging with NAC significantly reduced the LPA-induced expression of ChREBP and fibrotic factors in SV40 MES13 cells, suggesting that the production of ROS by LPA contributes to the ChREBP-mediated mesangial fibrosis.

We observed that LPA significantly increased the expression of ChREBP mRNA in SV40 MES13 cells; however, the change in ChREBP expression following transcriptional inhibition with actinomycin D was relatively low. This result suggests that the increase of LPA-induced ChREBP protein expression is primarily mediated via protein stabilization mechanisms. We therefore investigated the mechanism underlying the stabilization of ChREBP protein. Smurf2 is a HECT-type E3 ubiquitin ligase that was initially identified as a regulator of Smad protein stability in the TGF-β/bone morphogenetic protein (BMP) signaling pathway [54]. Apart from the regulation of TGF-β signaling, multiple biological functions of Smurf2 have been reported, including its role in genome stability, cell polarity, cell cycle, tissue homeostasis, and tumorigenesis [15, 44]. According to a recent report [27], Smurf2 inhibits the proliferation of cancer cells by promoting the ubiquitination and degradation of ChREBP. In this study, Heclin was used to inhibit the activity of Smurf2. Heclin can clearly distinguish between RING- and HECT-mediated ubiquitination as it specifically inhibits the activity, but not the expression, of HECT-type E3 ubiquitin ligases [28]. Consistent with a previous report [28], we observed that treatment with Heclin did not affect the expression of Smurf2; however, the expression of the target factor, ChREBP, increased when the E3 ligation of Smurf2 was inhibited. Our results demonstrated that LPA decreased the expression of Smurf2 and subsequently reduced the ubiquitination of ChREBP in SV40 MES13 cells. Further analyses revealed that the overexpression of Smurf2 increased the ubiquitination of ChREBP and decreased the expression of ChREBP protein in SV40 MES13 cells. In the present study, we showed total ubiquitination of ChREBP. Based on a recent report that Smurf2 mediates K48-linked ubiquitination of PTX3 [55], we expect that ChREBP also has K48-linked ubiquitination. Further studies are needed to accurately determine the ubiquitinated bands of ChREBP. Meanwhile, we observed that the pharmacological inhibition or knockdown of Smurf2 in SV40 MES13 cells not only increased the protein expression of ChREBP, but also increased the expression of fibrotic factors. Additionally, we observed that the glomerular expression of Smurf2 was decreased in db/db mice; however, it was restored in db/db mice treated with ki16425. Similar to our results, it has been previously reported that the activity of Smurf2 is decreased in human fibrotic liver, and that the hepatic overexpression of Smurf2 attenuates liver fibrosis [56], indicating that the expression of Smurf2 might play a role in the inhibition of fibrotic response.

In addition to ubiquitination, the stability of the ChREBP protein is affected by O-GlcNAcylation, a regulatory post-translational modification [16, 57]. As O-GlcNAcylation increases the expression and stability of proteins, and reduces protein ubiquitination [57], it is possible that the O-GlcNAcylation of ChREBP might play a role in the suppression of ChREBP ubiquitination by LPA in mesangial cells. According to a previous report, the O-GlcNAcylation of ChREBP increases in mesangial cells exposed to high levels of glucose, which induces fibrosis [16]. It has been additionally reported that the increased O-GlcNAcylation of ChREBP in hepatocytes leads to decreased ubiquitination during lipogenesis [58]. These results indicate that LPA might also induce the O-GlcNAcylation of ChREBP for increasing the expression of ChREBP protein in mesangial cells. Further studies are necessary for defining the roles of O-GlcNAcylation and ubiquitination of ChREBP in LPA-mediated diabetic renal fibrosis.

We subsequently investigated the changes in the expression of the signaling molecules that partake in the LPA-induced reduction of Smurf2 expression. LPA activates numerous intracellular signaling mediators to induce various cellular responses, including cellular proliferation and fibrosis [5, 59]. The LPA-induced production of ROS was demonstrated by the activation of the extracellular signal–regulated kinase (ERK), Akt, and nuclear factor kappa B (NF-κB) signaling pathways, leading to the increased proliferation of SKOV3 ovarian cancer cells [26]. We also observed that LPA induced the phosphorylation of Akt in mesangial cells, which was inhibited by treatment with NAC, an ROS scavenger. Furthermore, it has been recently reported that Akt inhibits the expression of Smurf2 in colorectal cancer cell lines [27]. We therefore examined whether the LPA-induced phosphorylation of Akt can regulate the expression of Smurf2 in mesangial cells. Akt is the most important mediator of various targets that receive the signals generated by PI3K [60]. The results of this study demonstrated that the pharmacological inhibition of Akt signaling by Ly 294002, a PI3K inhibitor, or A6730, an Akt inhibitor, restored the expression of Smurf2 that was reduced by LPA, and suppressed the LPA-induced expression of ChREBP. We also observed that LPA decreased the expression of Smurf2 protein without reducing the mRNA expression of Smurf2, suggesting that LPA reduced the stability of Smurf2 protein. According to previous reports, the downregulation of Smurf2 via Akt phosphorylation is attributed to the increased ubiquitination of Smurf2 [27, 29], and that RING domain E3 ubiquitin ligases, including Traf4 and Trib3, regulate Smurf2 ubiquitination [30]. Consistent with these reports, LPA significantly increased the expression of Traf4 and the ubiquitination of Smurf2 in SV40 MES13 cells, whereas ki16425 inhibited the changes induced by LPA. These results suggest that LPA decreases the expression of Smurf2 protein via the increased ubiquitination of Smurf2 in mesangial cells. Consistent with the results in LPA-treated mesangial cells, the glomerular expression of Smurf2 also decreased in db/db mice, but was inhibited by administration of ki16425. All these results suggested that the activation of Akt by the LPA-induced production of ROS induces a fibrotic response by downregulating Smurf2 and increasing the expression of ChREBP in renal mesangial cells.

We previously observed that LPA increases the expression of TGF-β1 by inducing the activation of sterol regulatory element binding protein (SREBP)1 [10]. In the present study, we observed that the increased expression of ChREBP contributed to the induction of fibrotic factors, such as TGF-β1. It has been reported that the increase in the expression and activity of SREBP-1 and ChREBP in the kidneys of mice with type 1 diabetes together possibly playing a role in the increase of fibrosis factor by fatty acid synthesis [45]. It is therefore possible that the interaction between SREBP1 and ChREBP synergistically induces the expression of fibrotic factors in renal mesangial cells by LPA. Further studies are necessary for elucidating the detailed underlying molecular mechanisms.

## Conclusions

**Fig. 10:**
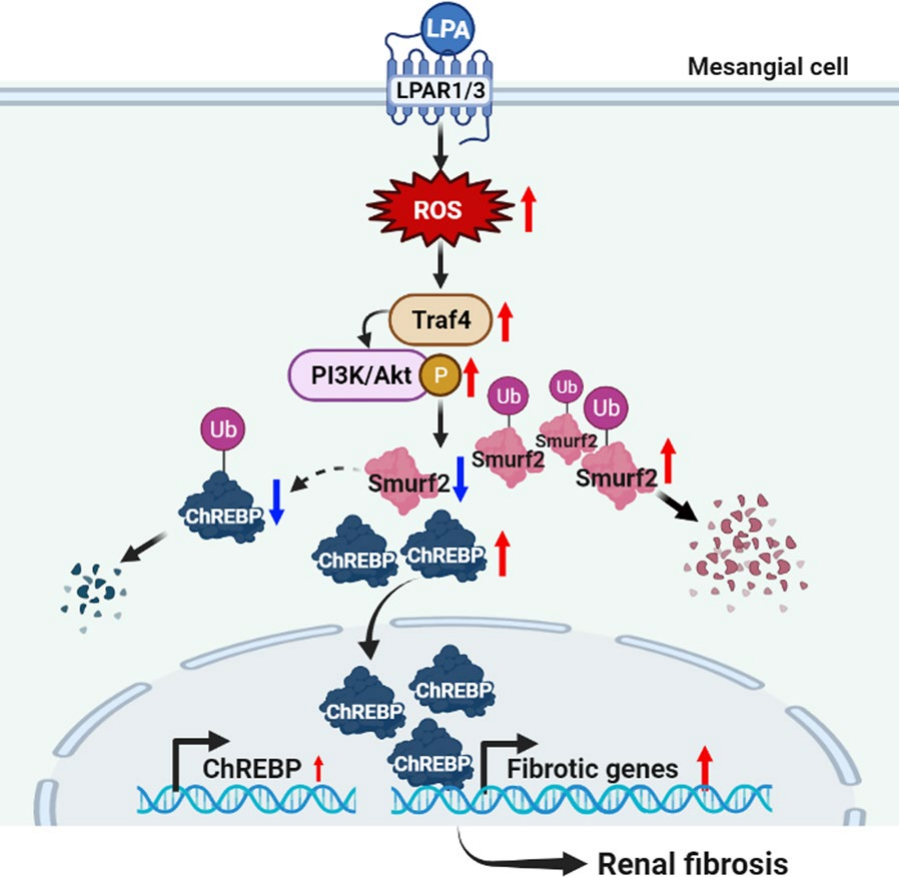
Schematic representation of the mechanism by which LPA induces ChREBP-mediated fibrosis, via the ROS/Akt-dependent downregulation of Smurf2 in renal mesangial cells. Created with BioRender.com. Red and blue arrows indicate upregulated and downregulated responses, respectively

Collectively, the results of this study demonstrated that the LPA-induced production of ROS downregulated Smurf2 in a Traf4 and Akt phosphorylation-dependent manner, which in turn decreased the ubiquitination of ChREBP (Fig. 10). This, in part, resulted in an increased expression of ChREBP, leading to a fibrotic response in mesangial cells. The pathway described herein could be one of the mechanisms underlying LPA-induced fibrosis in mesangial cells, and might provide a potential novel target for the development of therapeutic strategies against DN.

## Supplementary Information

**Additional file 1: Fig. S1.** Glomerular expression of ChREBP and α–smooth muscle actin (α-SMA) in wild type mice (related to Fig. 1C). **Fig. S2.** The LPA-induced expression of ChREBP protein is partially mediated via transcription in SV40 MES13 cells. **Fig. S3.** Transfection efficiency of ChREBP and Smurf2 overexpression in SV40 MES13 cells. **Fig. S4.** ChREBP mediates the LPA-induced proliferation of SV40 MES13 cells. **Table S1.** Primers used for quantitative real-time PCR.

## Data Availability

The datasets used and analyzed during the current study are available from the corresponding author on reasonable request.

## Abbreviations

AGEs: Advanced glycation end products
ANOVA: One-way analysis of variance
bHLH-LZ: Basic helix–loop–helix leucine zipper
ChREBP: Carbohydrate-responsive element-binding protein
FAF-BSA: Fatty acid-free bovine serum albumin
GPCRs: G protein-coupled receptors
Ki: Ki16425
LPA: Lysophosphatidic acid
LPC: Lysophosphatidyl choline
LPAR: LPA receptor
NAC: N-acetyl-l-cysteine
qRT-PCR: Quantitative real-time polymerase chain reaction
RAS: Renin-angiotensin system
ROS: Reactive oxygen species
SEM: Standard error of the mean
SFM: Serum-free medium
SGLT2: Sodium-glucose co-transporter 2
siRNA: Small interfering RNA
Smurf2: Smad ubiquitination regulatory factor-2
SREBP: Sterol regulatory element binding protein
STZ: Streptozotocin
SV40 MES13: SV40-transformed murine glomerular mesangial cells
