# Supplementary material for: Inhibition of ChREBP ubiquitination via the ROS/Akt-dependent downregulation of Smurf2 contributes to lysophosphatidic acid-induced fibrosis in renal mesangial cells
Source: J Biomed Sci. 2022 May 10;29:31. doi: 10.1186/s12929-022-00814-1 (PMC9092836; doi:10.1186/s12929-022-00814-1)
Supplement: Supplementary file 1 — Additional file 1: Fig. S1. Glomerular expression of ChREBP and α–smooth muscle actin (α-SMA) in wild type mice (related to Fig. 1C). Fig. S2. The LPA-induced expression of ChREBP protein is partially mediated via transcription in SV40 MES13 cells. Fig. S3. Transfection efficiency of ChREBP and Smurf2 overexpression in SV40 MES13 cells. Fig. S4. ChREBP mediates the LPA-induced proliferation of SV40 MES13 cells. Table S1. Primers used for quantitative real-time PCR. [file 12929_2022_814_MOESM1_ESM.docx]

**Supplementary data**


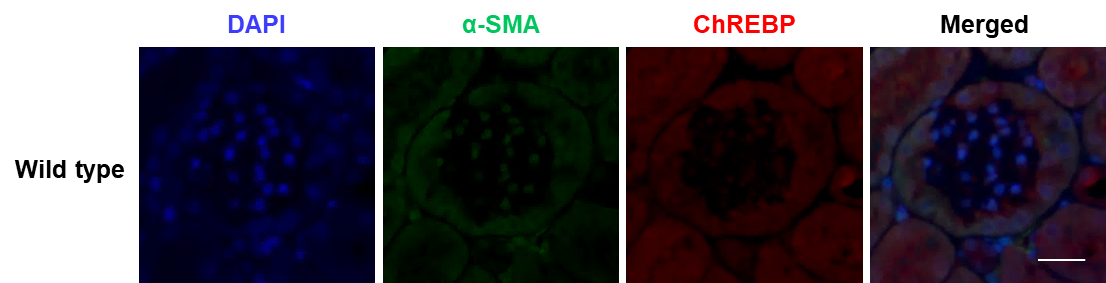


**Additional file 1: *Fig. S1. Glomerular expression of ChREBP and α–smooth muscle actin (α-SMA) in wild type mice (related to Fig. 1C).*** Representative images depicting the expression of α-SMA (green) and ChREBP (red) in the kidney tissue sections of wild type mice. The nuclei were counterstained with DAPI (blue). Scale bar, 20 μm; n=3.


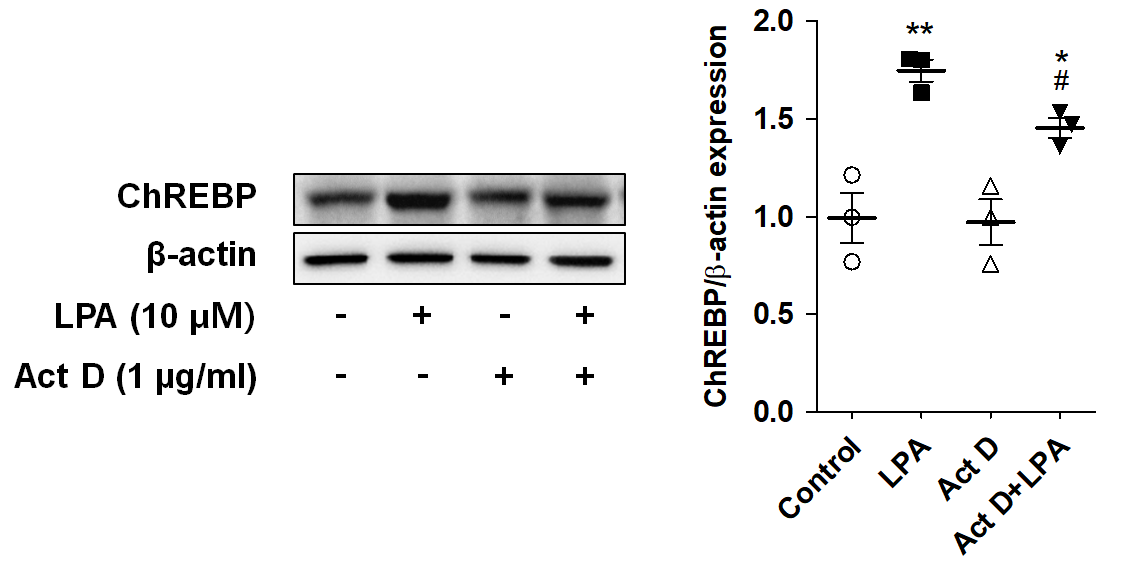


**Additional file 1: Fig. S2. The LPA-induced expression of ChREBP protein is partially mediated via transcription in SV40 MES13 cells.** SV40 MES13 cells were pretreated with actinomycin D (Act D, 1 μg/ml) for 1 h, and subsequently treated with LPA for 3 h. The protein level of ChREBP was analyzed by western blotting, quantified using ImageJ software, and normalized to that of β-actin. The data are presented as the mean ± SEM of results obtained from three independent experiments. *p<0.05, **p<0.01 vs. vehicle only (control); #p<0.05 vs. LPA only.


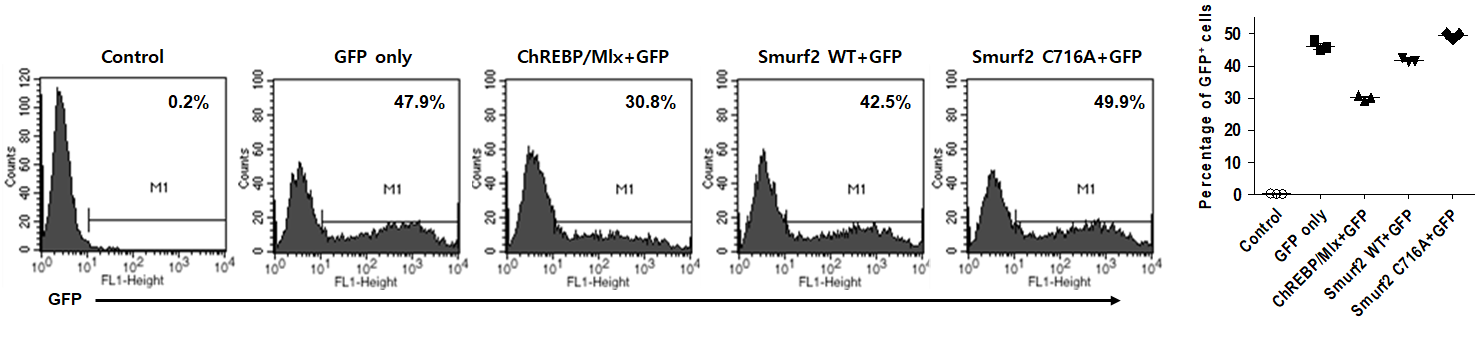


**Additional file 1: Fig. S3. Transfection efficiency of ChREBP and Smurf2 overexpression in SV40 MES13 cells.** SV40 MES13 cells were seeded at a density of 3 × 10^5^ cells/60-mm dish, 24 h prior to transfection. In order to determine the transfection efficiency of ChREBP or Smurf2 overexpression, the cells were transiently co-transfected with the enhanced green fluorescent protein (EGFP) and ChREBP/Mlx, EGFP and Smurf2 wt, or EGFP and Smurf2 C716A expression vectors using Lipofectamine 3000, according to the manufacturer’s instructions. The GFP-positive cells were quantified by flow cytometry after 24 h of transfection. The values in the representative flow cytometry histograms indicate the percentage of GFP-positive cells in the total cell population. The data are presented as the mean ± SEM of results obtained from three independent experiments.


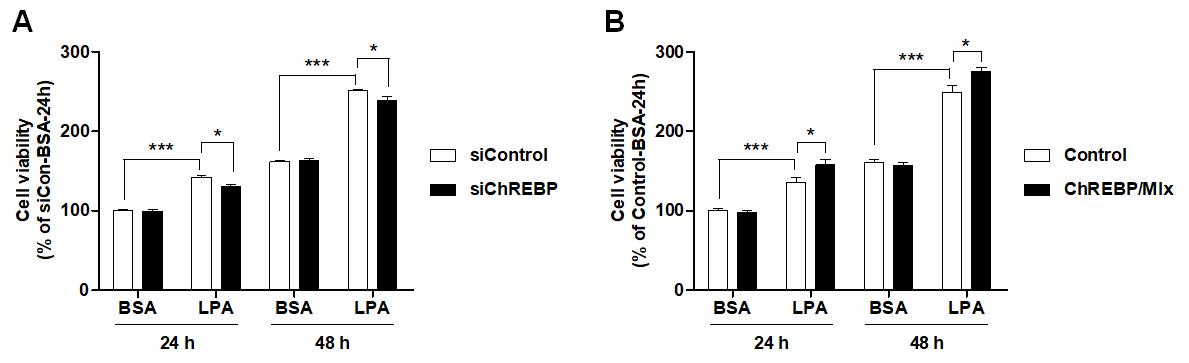


**Additional file 1: Fig. S4. ChREBP mediates the LPA-induced proliferation of SV40 MES13 cells.** (A) SV40 MES13 cells were transfected with a control siRNA (siCon) or ChREBP siRNA (siChREBP) and treated with LPA for 24 or 48 h. Cell viability was analyzed by CCK8 assay and calculated as a percentage relative to that of siCon-BSA cells at 24 h. (B) The SV40 MES13 cells were co-transfected with vectors expressing ChREBP and Mlx (ChREBP/Mlx), or transfected with an empty pcDNA vector (Control), and treated with LPA for 24 or 48 h. Cell viability was analyzed by CCK8 assay and calculated as a percentage relative to that of the Control-BSA cells at 24 h. The data are presented as the mean ± SEM of results obtained from three independent experiments. *p<0.05, **p<0.01, ***p<0.005.

**Additional file 1: Table S1. Primers used for quantitative real-time PCR**

| Gene name | Sequences 5′-3′ |
| --- | --- |
| *ChREBP* | Forward 5′-AGAACCGACGTATCACACACATCT-3′ |
|  | Reverse 5′-CAGGGTGTCGAATCCTAGCTTAA-3′ |
| *Fibronectin* | Forward 5′-TGACGATGGGAAGACCTACCA-3′ |
|  | Reverse 5′-GGAACAAATGGCTCCGAGATAT-3′ |
| *TGF-β* | Forward 5′-GCAGTGGCTGAACCAAGGA-3′ |
|  | Reverse 5′-AGCAGTGAGCGCTGAATCG-3′ |
| *IL-1β* | Forward 5′-CTACAGGCTCCGAGATGAACAAC-3′ |
|  | Reverse 5′-TCCATTGAGGTGGAGAGCTTTC-3′ |
| *Smurf2* | Forward 5′-TGGGGACATGTCTAACCCC-3′ |
|  | Reverse 5′-TTTTGCACAGAGTACTGTCAGG-3′ |
| *Traf4* | Forward 5′-GAGCCTGTGCAGGTGTCTAC-3′ |
|  | Reverse 5′-AGACTCCTTCACTGAGAAACTCC-3′ |
| *Trib3* | Forward 5′-CAGCAACTGTGAGAGGACGA-3′ |
|  | Reverse 5′-AGTCATCACGCAGGCATCTT-3′ |
| *Ttc3* | Forward 5′-CCATAGCAGGCGCAATTCAG-3′ |
|  | Reverse 5′-AACATACTCGCTGCTGTGCT-3′ |
| *Usp11* | Forward 5′-CCACGCATACAAGTGTTGCACC-3′ |
|  | Reverse 5′-CTCAATCCGACCAGTCACCTCA-3′ |
| *18s rRNA* | Forward 5′-CACTACGCGCAGTATGTGCT-3′ |
|  | Reverse 5′-TGAATGGCCTCCTTATCCTG-3′ |
